# Association Between Mefox and Prevalence of COPD: Evidence from NHANES 2011–2020

**DOI:** 10.3390/healthcare14010040

**Published:** 2025-12-23

**Authors:** Long Liang, Ying Luo, Yongchang Sun

**Affiliations:** Department of Respiratory and Critical Care Medicine, Peking University Third Hospital, Beijing 100191, China; luoying@stu.pku.edu.cn

**Keywords:** COPD, Mefox, logistic regression model, RCS regression model, inflammatory

## Abstract

**Objective**: This study aimed to investigate the relationship between Mefox (a pyrazino-s-triazine derivative of 4α-hydroxy-5-methyl-tetrahydrofolate and an oxidative stress marker derived from folate metabolism) levels and the prevalence of Chronic Obstructive Pulmonary Disease (COPD) using data from the National Health and Nutrition Examination Survey (NHANES) collected between 2011 and 2020. **Methods**: The analysis included 9525 participants after excluding those with incomplete data. COPD was defined through self-reported diagnoses, while Mefox levels were measured using HPLC-MS/MS techniques. Logistic regression analyses were conducted to assess the association between Mefox and COPD prevalence, with adjustments for demographic and clinical covariates. Restricted cubic spline (RCS) regression models were employed to explore the dose–response relationship. **Results**: The weighted prevalence of COPD was found to be 4.0%. Higher Mefox levels were significantly associated with increased COPD risk (*p* = 0.00117, odds ratio [OR] 1.72, 95% confidence interval [CI] 1.24–2.39). A nonlinear association was observed, with risk stability at lower Mefox levels, followed by a significant increase at higher levels. Subgroup analyses revealed consistent associations across demographics, with significant interactions noted in age > 70 years adults and individuals with BMI ≥ 25. Additionally, inflammatory markers showed significant correlations with Mefox levels. **Conclusions**: The findings highlight a significant association between elevated Mefox levels and increased COPD risk, suggesting that disruptions in folate metabolism and inflammation may play crucial roles in COPD pathogenesis. These results underscore the need for further research to explore potential therapeutic interventions targeting folate metabolism in COPD management.

## 1. Introduction

Chronic Obstructive Pulmonary Disease (COPD) is a leading cause of global morbidity and mortality, characterized by persistent respiratory symptoms and progressive, irreversible airflow limitation [1]. COPD places a substantial burden on healthcare systems [2], with primary risk factors including smoking, environmental pollutants, and occupational exposures. Despite these well-established contributors, emerging evidence suggests that oxidative stress [3,4] plays a crucial role in the development and progression of COPD. Oxidative stress occurs when there is an imbalance between the production of reactive oxygen species (ROS) and the body’s antioxidant defenses, leading to cellular damage, inflammation, and the gradual decline in lung function observed in COPD patients.

The potential connection between folate metabolism [5] and COPD has gained increasing attention, as folate is essential for DNA synthesis, repair [6], and methylation [7]. Mefox (a pyrazino-s-triazine derivative of 4α-hydroxy-5-methyl-tetrahydrofolate) is an oxidation product of 5-methyl-tetrahydrofolate (5-mTHF), a critical form of folate. When oxidative stress occurs in the body, 5-mTHF can attenuate oxidative stress to form Mefox. Elevated levels of Mefox indicate oxidative damage and impairments in folate metabolism, which may contribute to the pathogenesis of various chronic diseases, including cardiovascular disorders [8] and cancer [9].

While the relationship between folate deficiency and COPD is still under investigation, folate is believed to play a role in maintaining lung health, as it helps regulate homocysteine levels [10], which are associated with oxidative stress and inflammation. Elevated homocysteine levels are linked to the severity of COPD [11]. Therefore, the relationship between Mefox and oxidative stress provides a potential mechanistic link between folate metabolism and COPD. Understanding this connection is crucial, as oxidative stress not only contributes to the onset of COPD but also exacerbates its progression [12], leading to more severe respiratory symptoms and faster decline in lung function.

The National Health and Nutrition Examination Survey (NHANES) data from 2011 to 2020 offer a valuable resource for examining the health and nutritional well-being of the U.S. population. Leveraging this dataset provides a unique opportunity to explore the potential link between Mefox and the prevalence of COPD. This study intends to investigate whether elevated Mefox levels, which serve as markers of oxidative stress and disruptions in folate metabolism, are correlated with an increased risk of COPD diagnosis.

## 2. Materials and Methods

### 2.1. Study Population and Design

**Figure 1:**
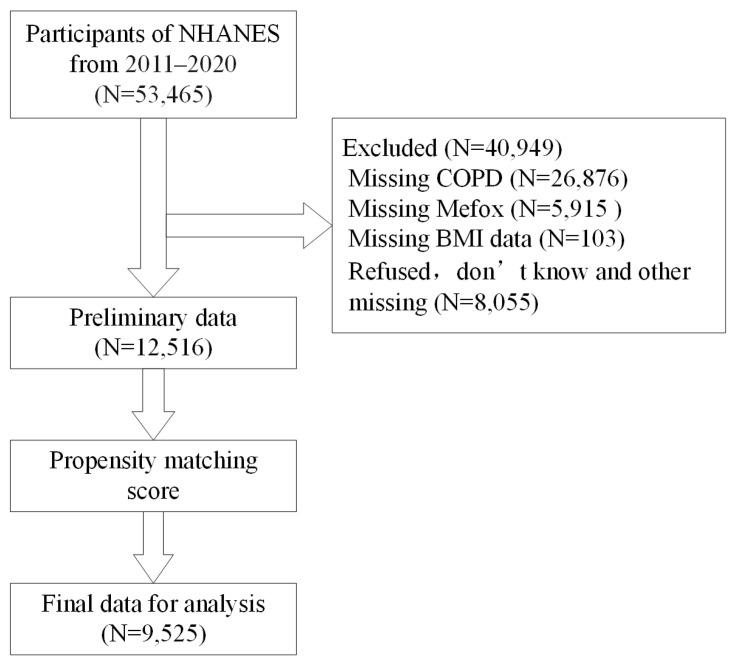
Flowchart of the participant enrollment and screening process for this study. NHANES, National Health and Nutrition Examination Survey; Mefox, pyrazino-s-triazine derivative of 4α-hydroxy-5-methyltetrahydrofolate.

The data in NHANES are implemented by the CDC’s NCHS, which assesses the health and nutritional status of the U.S. ambulatory population. It uses a stratified, multi-stage probability sampling design that combines household interviews with medical and physical examinations at mobile examination centers (MECs). Data are publicly available on the official website (https://www.cdc.gov/nchs/nhanes/ (accessed on 20 February 2024)). The NCHS Research Ethics Review Board approved the survey, and all participants provided informed consent. This study included 53,465 participants from the 2011–2020 NHANES cycles. After excluding those with missing COPD data (N = 26,876), missing Mefox data (N = 5915), missing BMI data (N = 103), refused, do not know and other missing data (N = 8055), the preliminary data (N = 12,516) was propensity score matched, and ultimately 9525 participants were included in the analysis (Figure 1).

### 2.2. Definition of COPD

According to previous research by Kavita et al. [13], the screening criteria for COPD patients utilized a self-reported method. The screening process involved three key self-reported survey questions: ‘Has a doctor ever told you that you have chronic bronchitis?’, ‘Has a doctor ever told you that you have emphysema?’, and ‘Has a doctor or other health professional ever informed you that you have COPD?’. These questions were designed to encompass the main types of COPD while also considering common respiratory diseases associated with it. If participants answered ‘yes’ to any of these questions, they were classified into the COPD group, indicating that they had relevant medical diagnoses and may have experienced symptoms and treatments related to the disease. Conversely, if participants answered ‘no’ to all questions, they were categorized as the non-COPD group. This classification method allows the study to more clearly delineate the health status of different groups, although it does not capture spirometry-defined airflow limitation and may be subject to recall and reporting bias.

### 2.3. Biomarker Measurement

Serum samples from participants aged 1 year and above were examined for Mefox using HPLC-MS/MS techniques in the CDC lab. This highly precise and sensitive method was utilized to measure Mefox, a folate metabolite that serves as an indicator of oxidative stress in the body. The study analyzed data from 9525 individuals, offering a robust dataset for exploring potential associations between Mefox concentrations and various health outcomes.

### 2.4. Covariates

The analysis included a comprehensive collection of covariate data across several domains. First, demographic characteristics were recorded, encompassing age, gender, ethnicity, education level, health insurance status, sedentary time, and past-year alcohol consumption. Additionally, participants were assessed for their smoking dose, specifically whether they had smoked 100 or more cigarettes in their lifetime. Second, physical examination parameters were captured, with a particular focus on body mass index (BMI), which serves as an important indicator of overall health. BMI was calculated by dividing weight in kilograms (kg) by height in meters squared (m^2^) and classified into two categories: those with a BMI of less than 25 kg/m^2^ and those with a BMI of 25 kg/m^2^ or higher, indicating potential overweight or obesity. Third, a range of medical conditions was documented, including high cholesterol levels, diabetes, hypertension, coronary heart disease, stroke, and cancer. These conditions are significant as they can influence the overall health status and risk factors for various diseases. Finally, laboratory findings were extensively evaluated, which included measurements of white blood cell counts, lymphocytes, monocytes, neutrophils, eosinophils, basophils, red blood cells, hemoglobin, hematocrit, mean cell volume, mean cell hemoglobin concentration, red cell distribution width, platelet counts, and alkaline phosphatase (ALP) levels. Collectively, this data provides a robust framework for understanding the health profile of the participants and facilitates a deeper investigation into the associations between these covariates and the outcomes of interest.

### 2.5. Statistical Analysis

Due to the complexity of the NHANES sample questionnaires, we employed sample weighting and stratification to correct for potential biases. Categorical variables were analyzed using chi-square tests. The study first assessed the relationship between Mefox levels and COPD prevalence using univariate logistic regression, followed by multivariate logistic regression analysis. The primary outcome variable was a binary indicator of COPD (1 = COPD, 0 = non-COPD), defined according to self-reported physician diagnosis. All logistic regression models estimated the odds of COPD as the response variable. To further investigate the dose–response relationship between Mefox and COPD risk, we utilized a restricted cubic spline regression (RCS) model. Additionally, subgroup analyses were conducted to confirm the association between Mefox levels and COPD across different demographic groups. We also evaluated the relationship between Mefox levels and various blood cell parameters through multivariable linear regression and conducted mediation analysis to explore whether inflammation serves as a key mediator in this association. The mediators included white blood cell count, lymphocyte count, monocyte count, segmented neutrophil count, eosinophil count, basophils, red blood cell count, hemoglobin, mean cell volume, mean cell hemoglobin concentration, red cell distribution width, and alkaline phosphatase (ALP). Mediation analyses were implemented using a nonparametric bootstrapping framework (Imai, Keele, & Tingley) [14], which generated estimates of the total, direct, and indirect effects and their bias-corrected 95% confidence intervals. For continuous variables following a normal distribution, independent-samples *t*-tests were performed. For skewed variables, including Mefox, non-parametric Mann–Whitney U tests were applied. All statistical analyses were performed using R software (version 4.4.1), with a *p*-value < 0.05 considered statistically significant.

## 3. Results

### 3.1. Baseline Characteristics of Participants

**Figure 2:**
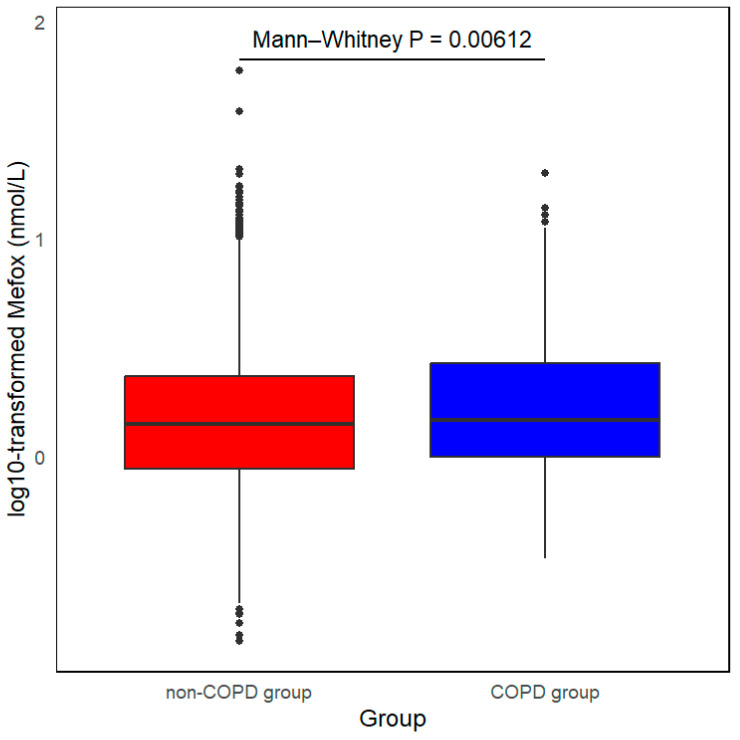
Comparison of log10-transformed Mefox levels in the non-COPD and COPD groups. *p* = 0.00612, as compared with non-COPD group. Mefox, pyrazino-s-triazine derivative of 4α-hydroxy-5-methyltetrahydrofolate; COPD, chronic obstructive pulmonary disease. Dots represent individual observations lying outside the whisker range (1.5× interquartile range).

The global prevalence of COPD was statistically 3–6% [15] in 1990 and 2017. This study included 9525 participants from the NHANES database collected between 2011 and 2020. The weighted prevalence of COPD was calculated at 4.0%, consisting of 381 participants diagnosed with COPD and 9144 without. Figure 2 illustrates that individuals with COPD had significantly higher levels of Mefox compared to their non-COPD counterparts (*p* = 0.00612). Participants were stratified into four groups based on quartiles of log10-transformed Mefox levels: Q1 (log10 Mefox < −0.06 nmol/L, n = 2405), Q2 (−0.06 nmol/L ≤ log10 Mefox < 0.15 nmol/L, n = 2366), Q3 (0.15 nmol/L ≤ log10 Mefox < 0.37 nmol/L, n = 2389), and Q4 (log10 Mefox ≥ 0.37 nmol/L Hb, n = 2365). More information can be found in Table 1.

### 3.2. Association Between Mefox and COPD

Table 2 indicates that univariate logistic regression analysis found a significant link between log10-transformed Mefox levels and an elevated risk of COPD (odds ratio [OR] 1.72, 95% confidence interval [CI] 1.24–2.39, *p* = 0.00117). Chi-square tests revealed significant associations with several variables, including log10-transformed Mefox, age, education level, sedentary behavior, alcohol use, smoking, high blood pressure, coronary heart disease, and stroke. As indicated in Table S1. Univariate variables that were significant in the above chi-square test were included in multivariate logistic regression model I. The covariates included in the regression model were age, education level, sedentary behavior, alcohol use, smoking, high blood pressure, coronary heart disease, and stroke. Several covariates showed significant associations with COPD. Increasing age (OR = 1.23, 95% CI: 1.12–1.34, *p* = 5.13 × 10^−6^) was positively associated with COPD prevalence, whereas higher education level (OR = 0.88, 95% CI: 0.81–0.96, *p* = 0.005929) was inversely associated. Smoking (OR = 0.29, 95% CI: 0.22–0.37, *p* < 0.0001), coronary heart disease (OR = 0.60, 95% CI: 0.42–0.84, *p* = 0.004082), and stroke (OR = 0.50, 95% CI: 0.35–0.73, *p* = 0.000257) were also significantly associated with COPD risks. Supplementary Materials can be found in Tables S2 and S3.

**Figure 3:**
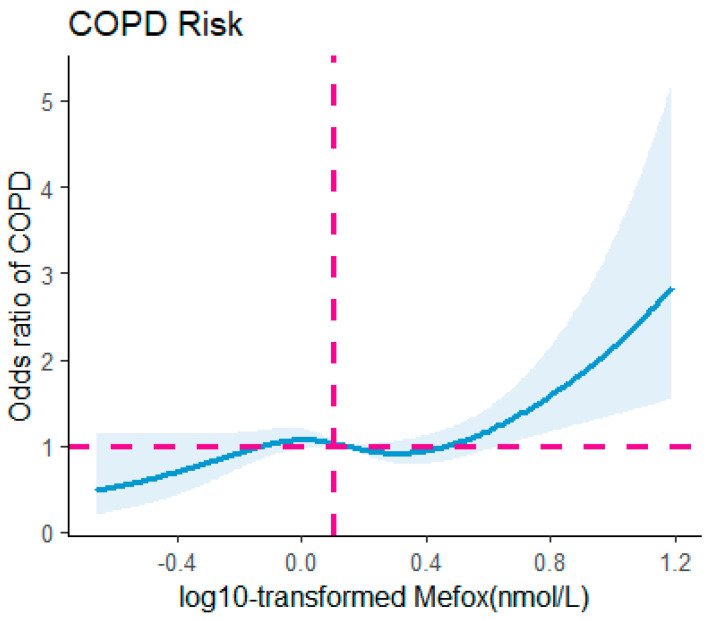
The odds ratio for COPD was analyzed concerning log10-transformed Mefox levels in the overall population. A nonlinear relationship was detected (*p* for nonlinearity = 0.0305) through a restricted cubic spline regression (RCS) model. The solid line illustrates the odds ratio for COPD, while the shaded area indicates the 95% confidence interval. COPD, chronic obstructive pulmonary disease; Mefox, pyrazino-s-triazine derivative of 4α-hydroxy-5-methyltetrahydrofolate. Dashed lines indicate the odds ratio (OR = 1.0, horizontal) and the reference level of log10-transformed Mefox (vertical).

Figure 3 illustrates that the RCS model revealed a nonlinear association between log10-transformed Mefox levels and COPD risk after adjusting for confounding variables (*p* for nonlinearity = 0.0305). The risk of COPD showed relative stability for log10-transformed Mefox levels in the range of 0–0.4 nmol/L, after which a significant rise was observed.

### 3.3. Subgroup Analysis

**Figure 4:**
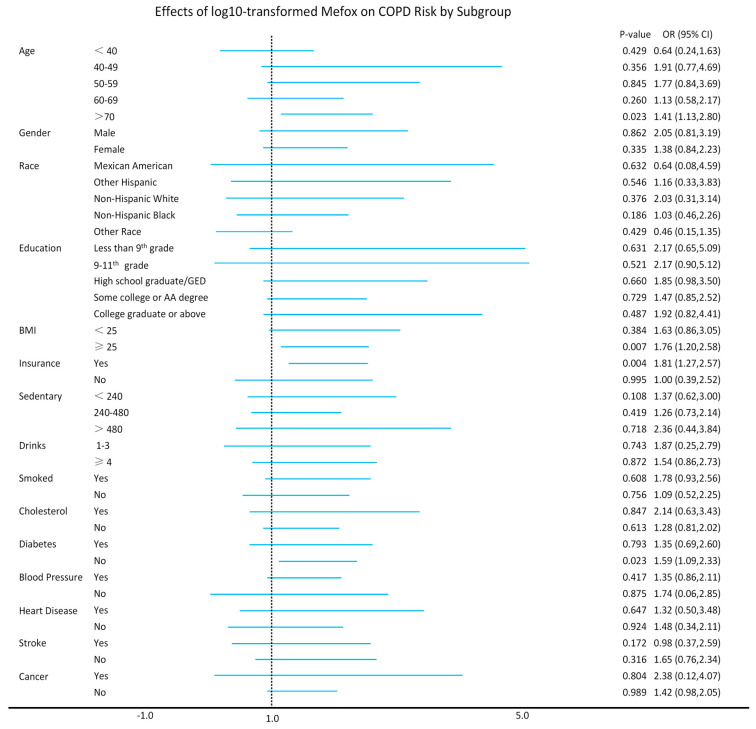
The association between log10-transformed Mefox levels and COPD risk across various subgroups. Mefox, pyrazino-s-triazine derivative of 4α-hydroxy-5-methyltetrahydrofolate; COPD, chronic obstructive pulmonary disease; OR, odds ratio; CI, confidence interval; BMI, body mass index.

The subgroup analysis examined whether the association between log10-transformed Mefox levels and the risk of COPD varied across different groups. As shown in Figure 4, no significant interaction was found when stratified by variables such as gender, race, education level, sedentary time, past year alcohol drinking, smoked 100 cigarettes, high cholesterol level, high blood pressure, coronary heart disease, stroke, and cancer. These findings suggest that the association between log10-transformed Mefox levels and COPD risk remains consistent across these subgroups, with no evidence of effect modification (*p* for interaction > 0.05). However, a significant interaction was observed when stratifying by age > 70 years (*p* = 0.023), BMI ≥ 25 (*p* = 0.007), health insurance status (*p* = 0.004), and without diabetes (*p* = 0.023).

### 3.4. Association Between Mefox and Inflammatory Factors

A multivariate linear regression analysis was conducted to explore the relationship between Mefox and inflammatory markers. As shown in Table 3, log10-transformed Mefox levels exhibited a significant positive correlation with white blood cells (β = 0.11067; *p* = 0.0243), lymphocytes (β = −0.1172; *p* = 0.0170), eosinophils (β = 0.1777; *p* = 0.0006), and ALP (β = 0.0003; *p* = 0.0236). These findings suggest that Mefox is linked to inflammation. Additionally, we found that log10-transformed Mefox levels had a significantly positive association with red blood cells (β = 0.1148; *p* = 0.0201), hemoglobin (β = −0.0674; *p* = 5.61 × 10^−5^), mean cell volume (β = 0.0082; *p* = 0.0020), and mean cell hemoglobin concentration (β = 0.0483; *p* = 4.10 × 10^−10^). This suggests that log10-transformed Mefox levels are closely related to anemia and oxygenation status. Understanding changes in Mefox levels can help doctors better assess patients’ blood health and may provide new insights for managing respiratory diseases like COPD, ultimately improving patient outcomes.

### 3.5. Inflammatory Factors Involved in the Effects of Mefox on COPD

To further examine how inflammatory and hematologic markers may contribute to the association between Mefox and COPD, we conducted mediation analyses (Table 4). Several markers, including white blood cells, lymphocytes, segmented neutrophils, eosinophils, basophils, and ALP, showed statistically significant indirect effects, indicating that a meaningful proportion of the Mefox–COPD association may be accounted for by these inflammatory pathways. For example, white blood cell count displayed a substantial indirect effect (β_indirect = 1.3181, 95% CI 1.0038–1.6323), while the direct association of Mefox with COPD remained smaller (β_direct = 0.0211, 95% CI 0.0083–0.0339), yielding a total effect of 1.3392 (95% CI 1.0247–1.6537). Similar patterns were observed for segmented neutrophils (β_indirect = 1.1057, 95% CI 0.9953–1.2161) and ALP (β_indirect = 3.8530, 95% CI 2.3387–5.3673).

In addition, several red blood cell indices, such as red blood cell count, hemoglobin, mean cell volume, mean cell hemoglobin concentration, and red cell distribution width, also exhibited significant indirect and total effects, suggesting that alterations in erythropoiesis and oxygen-carrying capacity may be statistically related to the Mefox–COPD association. Although some markers, such as monocyte count, did not demonstrate statistically significant indirect effects, the overall pattern supports a role for systemic inflammation and hematologic alterations in shaping the Mefox–COPD relationship.

## 4. Discussion

This research explored the relationship between Mefox and the prevalence of COPD utilizing data from the NHANES collected between 2011 and 2020. By examining Mefox levels as a biomarker for disturbances in folate metabolism, our research identified a significant positive correlation between elevated Mefox levels and COPD prevalence. RCS model demonstrated a nonlinear relationship between Mefox levels and COPD risk, indicating that risk remains relatively stable at lower Mefox concentrations but increases sharply at higher levels. These findings underscore the potential utility of Mefox as a biomarker for the effective identification of individuals at increased risk for COPD.

Subgroup analyses revealed that the association between Mefox and COPD was consistent across most demographic and clinical groups. Notably, we observed significant interactions in certain subgroups, particularly among participants aged 70 and older, those with a BMI ≥ 25, individuals with health insurance, and those without diabetes. These results suggest that age, obesity, and access to healthcare may modulate the strength of the association between Mefox and COPD risk. Pfeiffer [16] et al. reported a gradual increase in Mefox concentration distribution with advancing age, particularly higher detection rates in individuals over 60. Additionally, Zia Fazili’s research [17] found elevated Mefox levels in obese adults, smokers, and individuals with impaired kidney function or heightened inflammation. Liu et al. [18] used data from a large, nationally representative cohort of U.S. adults from 2011 to 2014, recruiting 10,661 patients tested for folate, and found a positive correlation between serum Mefox levels and all-cause/cardiovascular mortality. Interestingly, in our study, the presence of diabetes appeared to weaken the association between Mefox and COPD, potentially due to the complex interplay between diabetes and inflammation, suggesting that future research should explore how comorbidities like diabetes affect the relationship between Mefox and COPD.

BMI was included as a standardized anthropometric indicator available across all NHANES cycles. Although BMI does not distinguish between fat and lean mass and may misclassify muscular individuals, it remains widely used in COPD epidemiology as a marker of adiposity-related inflammation and metabolic burden. Because more specific measures of body composition were not uniformly available in the analytic sample, BMI was selected with acknowledgment of its limitations. The stronger association between Mefox and COPD observed among participants with BMI ≥ 25 also has important biological implications. Adipose tissue is now recognized as an endocrine-active organ that secretes a wide range of adipokines and pro-inflammatory cytokines, including tumor necrosis factor-α, interleukin-6, and leptin. Excess adiposity can therefore promote a chronic low-grade inflammatory state and increase systemic oxidative stress [19]. Under such conditions, folate-dependent one-carbon metabolism may be particularly vulnerable to oxidative degradation, leading to higher conversion of 5-mTHF to Mefox. Our findings are consistent with this model: in individuals with overweight or obesity, adipose tissue–derived inflammatory signals could amplify oxidative stress, elevate Mefox levels, and thereby strengthen the link between Mefox and COPD. This pattern underscores the importance of considering obesity, systemic inflammation, and folate metabolism jointly when evaluating COPD risk.

Our findings indicate that elevated Mefox levels are positively correlated with various inflammatory markers, such as leukocytes, eosinophils, and ALP. Research by Madhulika Tripathi [20] et al. has effectively demonstrated that vitamin B12 and folate can alleviate inflammation and fibrosis in non-alcoholic steatohepatitis. Additionally, Ajana Pathikkal [21] and colleagues reported that folate derivatives, such as 5-mTHF and 10-formyltetrahydrofolate (10-FTHF), can effectively combat high glucose (50 mM)-mediated oxidative stress and inflammation in bronchial epithelial cells (BEAS-2B). Mefox, as a metabolite of folate and 5-mTHF in antioxidant processes, serves as a valuable biomarker for both antioxidant activity and inflammation. Mediation analysis further revealed that specific inflammatory markers (such as leukocytes, neutrophils, and ALP) significantly mediate the relationship between Mefox and COPD prevalence. In a multivariable linear regression model constructed by Ziyi Liu [22], leukocytes (*p* = 0.002), ALP (*p* < 0.001), and C-reactive protein (*p* < 0.001) were negatively correlated with the Composite Dietary Antioxidant Index (CDAI). The study also confirmed that ALP and C-reactive protein influence the association between CDAI and COPD prevalence. These results highlight the key role of inflammation in the Mefox-COPD interaction, with the mediating role of eosinophils being particularly important because of its association with COPD exacerbation and severity. Surya *p* Bhatt [23] et al. reported that dupilumab effectively alleviates respiratory symptoms in COPD patients with elevated eosinophils, improving lung function and quality of life. Furthermore, Mefox levels are associated with changes in red blood cells, hemoglobin, and mean cell volume, suggesting a connection between anemia and oxygenation impairment in COPD patients. Ting Wang [24] included data from 308,592 participants in the UK Biobank to construct a polygenic risk score, calculating phenotypic age based on actual age and nine clinical biochemical markers, including albumin, ALP, creatinine, glucose, C-reactive protein, lymphocyte percentage, mean cell volume, red cell distribution width, and leukocyte count. The study assessed that PhenoAgeAccel is significantly associated with the risk of common chronic respiratory diseases, including COPD and decline in lung function. This further supports our findings that Mefox may be linked to oxygenation levels, exacerbating respiratory symptoms in COPD patients.

Our study highlights the significance of Mefox as a potential biomarker for COPD risk. Given the strong association between Mefox levels and COPD prevalence, measuring Mefox concentrations could provide clinicians with a valuable tool for assessing oxidative damage and predicting COPD risk, particularly in individuals exposed to other risk factors like smoking or environmental pollution. Moreover, since our findings suggest that inflammation mediates the impact of Mefox on COPD, targeting inflammatory mediators, especially eosinophils, may present new therapeutic strategies for managing the disease. Future research should focus on longitudinal studies to establish the causal relationship between Mefox and COPD development. Additionally, exploring the mechanisms that link Mefox and inflammatory responses could uncover new intervention targets. Considering the role of folate metabolism in Mefox production, examining the effects of folate supplementation or modulation on Mefox levels and COPD outcomes may also be worth investigating. An important conceptual issue is the possibility of reverse causality. Our analyses implicitly treat higher Mefox levels as an exposure that may increase the risk of COPD; however, it is also plausible that established COPD itself contributes to elevated Mefox concentrations. COPD is characterized by chronic airway and systemic inflammation, recurrent exacerbations, impaired gas exchange, and hypoxia, all of which can enhance oxidative stress. Increased oxidative stress, in turn, can accelerate the oxidation of 5-mTHF to Mefox. Under this scenario, Mefox levels would primarily reflect downstream consequences of COPD-related oxidative damage rather than a causal factor in disease onset.

This study has several important limitations. First, the cross-sectional nature of the NHANES data means that exposure and outcome were measured at the same time, which severely restricts our ability to draw causal inferences about the relationship between Mefox and COPD. Second, COPD was defined according to self-reported physician diagnoses of chronic bronchitis, emphysema, or COPD, rather than spirometry-defined airflow limitation (e.g., FEV_1_/FVC < 0.70 or below the lower limit of normal). Self-reported diagnosis is vulnerable to both underdiagnosis and misclassification. Third, the statistically significant group difference observed in Mefox levels should be interpreted in light of the large NHANES sample, which increases power to detect relatively small differences in distribution. Effect size estimates suggest a shift toward higher Mefox levels in the COPD group, but the clinical implications of this difference require cautious interpretation.

## 5. Conclusions

In summary, this study analyzed NHANES data and revealed a significant association between elevated Mefox levels and COPD, indicating that Mefox may serve as a potential biomarker for assessing COPD risk. This research also suggests that inflammation may play a crucial mediating role in the relationship between Mefox and COPD, particularly in older adults and individuals with obesity. These findings provide new insights for the early screening and intervention of COPD. Furthermore, further prospective studies and basic research are needed to validate our findings and to explore the role of Mefox in the pathogenesis of COPD and its potential clinical applications.

## Figures and Tables

**Table 1:** Characteristics of participants according to quartiles of log10-transformed Mefox.

Variables	Q1 Group (n = 2405)	Q2 Group (n = 2366)	Q3 Group (n =2389)	Q4 Group (n = 2365)	*p*-Value
Age (years)					<0.001
<40	640 (26.61)	534 (22.57)	464 (19.42)	401 (16.96)	
40–49	694 (28.86)	560 (23.67)	557 (23.32)	462 (19.53)	
50–59	507 (21.08)	505 (21.34)	499 (20.89)	449 (18.99)	
60–69	424 (17.63)	487 (20.58)	503 (21.05)	472 (19.96)	
≥70	140 (5.82)	280 (11.84)	366 (15.32)	581 (24.56)	
Gender, n (%)					0.0238
Male	1159 (48.19)	1140 (48.18)	1206 (50.48)	1227 (51.88)	
Female	1246 (51.81)	1226 (51.82)	1183 (49.52)	1138 (48.12)	
Ethnicity, n (%)					<0.001
Mexican America	318 (13.22)	220 (9.30)	200 (8.37)	182 (7.70)	
Other Hispanic	243 (10.10)	217 (9.17)	223 (9.33)	182 (7.70)	
Non-Hispanic White	707 (29.40)	1010 (42.69)	1139 (47.68)	1270 (53.70)	
Non-Hispanic Black	810 (33.68)	581 (24.56)	510 (21.35)	359 (15.18)	
Other Race	327 (13.60)	338 (14.28)	317 (13.27)	372 (15.72)	
Education level, n (%)					0.0071
<9th grade	147 (6.11)	135 (5.71)	128 (5.36)	140 (5.92)	
9–11th grade	304 (12.64)	238 (10.06)	208 (8.71)	229 (9.68)	
High school graduate/GED	511 (21.25)	496 (20.96)	523 (21.89)	501 (21.18)	
Some college or AA degree	764 (31.77)	766 (32.38)	803 (33.61)	758 (32.05)	
College graduate or above	679 (28.23)	731 (30.89)	727 (30.43)	737 (31.17)	
BMI (kg/m^2^)					<0.001
<25.0	719 (29.90)	664 (28.06)	610 (25.53)	577 (24.40)	
≥25.0	1686 (70.10)	1702 (71.94)	1779 (74.47)	1788 (75.60)	
Health insurance, n (%)					<0.001
Yes	1873 (77.88)	1971 (83.30)	2039 (85.35)	2058 (87.02)	
No	532 (22.12)	395 (16.70)	350 (14.65)	307 (12.98)	
Sedentary time (hours)					<0.001
<4 h/day	634 (26.36)	550 (23.25)	532 (22.27)	426 (18.01)	
4–8 h/day	915 (38.05)	936 (39.56)	926 (38.76)	909 (38.44)	
>8 h/day	856 (35.59)	880 (37.19)	931 (38.97)	1030 (43.55)	
Past-year alcohol drinking, n (%)					<0.001
1–3 drinks/day	1575 (65.49)	1619 (68.43)	1674 (70.07)	1742 (73.66)	
≥4 drinks/day	830 (34.51)	747 (31.57)	715 (29.93)	623 (26.34)	
Smoked 100 cigarettes in life, n (%)	1137 (47.28)	1101 (46.53)	1193 (49.94)	1213 (51.29)	0.002
High cholesterol, n (%)	801 (33.31)	880 (37.19)	977 (40.90)	1080 (45.67)	<0.001
Diabetes, n (%)	207 (8.61)	240 (10.14)	329 (13.77)	480 (20.30)	0.0005
Hypertension, n (%)	756 (31.43)	877 (37.07)	1001 (41.90)	1132 (47.86)	<0.001
Coronary heart disease, n (%)	35 (1.46)	72 (3.04)	113 (4.73)	161 (6.81)	<0.001
Stroke, n (%)	47 (1.95)	68 (2.87)	89 (3.73)	115 (4.86)	<0.001
Cancer, n (%)	166 (6.90)	222 (9.38)	265 (11.09)	373 (15.77)	<0.001

Note: The continuous log10-Mefox variable was categorized into quartiles based on its distribution in the entire analytic sample (COPD + non-COPD combined). Global quartiles were used to ensure consistent cutpoints across groups. MeFox, pyrazino-s-triazine derivative of 4α-hydroxy-5-methyltetrahydrofolate; BMI, body mass index.

**Table 2:** Multivariate logistic regression analysis of log10-transformed Mefox for risk of COPD.

	Crude Model		Model I	
	Crude OR (95%CI)	*p*-Value	Adjusted OR (95%CI)	*p*-Value
log10-Mefox	1.72 (1.24–2.39)	0.00117 **	1.15 (0.82–1.62)	0.420145
Q1 group	Reference		Reference	
Q2 group	1.45 (1.08–1.95)	0.01527 *	1.35 (1.0–1.84)	0.051906
Q3 group	1.06 (0.77–1.46)	0.71420	0.88 (0.64–1.22)	0.442394
Q4 group	1.55 (1.15–2.08)	0.00376 **	1.14 (0.84–1.55)	0.410152

Note: The crude model included all covariates listed in Table 1. Model I was adjusted for log10-transformed Mefox and covariates primarily selected from variables that were significantly associated in Table S1, including age, education level, sedentary behavior, alcohol use, smoking, high blood pressure, coronary heart disease, and stroke. MeFox, pyrazino-s-triazine derivative of 4α-hydroxy-5-methyltetrahydrofolate; OR, odds ratio; CI, confidence interval. * *p* < 0.05, ** *p* < 0.01.

**Table 3:** Multivariate linear regression of log10-transformed Mefox levels with inflammatory factors.

Variables	β	95%CI	*p*-Value
White blood cell (1000 cells/µL)	0.11067	1.437088 × 10^−2^, 0.206998973	0.0243
Lymphocytes (1000 cells/µL)	−0.1172	−2.135154 × 10^−1^, −0.020931362	0.017
Monocyte (1000 cells/µL)	0.0638	−3.708918 × 10^−2^, 0.164672562	0.2152
Segmented neutrophils (1000 cells/µL)	−0.0855	−1.820709 × 10^−1^, 0.011021844	0.0825
Eosinophils (1000 cells/µL)	0.1777	7.600135 × 10^−2^, 0.279425307	0.0006
Basophils (1000 cells/µL)	0.0165	−1.086711 × 10^−1^, 0.141650161	0.7962
Red blood cell (million cells/µL)	0.1148	1.802730 × 10^−2^, 0.211659422	0.0201
Hemoglobin (g/dL)	−0.0674	−1.001632 × 10^−1^, −0.034615532	5.61 × 10^−5^
Mean cell volume (fL)	0.0082	2.983943 × 10^−3^, 0.013371754	0.002
Mean cell Hgb concentration (g/dL)	0.0483	3.314787 × 10^−2^, 0.063392327	4.10 × 10^−10^
Red cell distribution width percent	0.0053	−3.856722 × 10^−4^, 0.011041446	0.0675
ALP (IU/L)	0.0003	4.048772 × 10^−5^, 0.000561878	0.0236

MeFox, pyrazino-s-triazine derivative of 4α-hydroxy-5-methyltetrahydrofolate; CI, confidence interval. ALP, alkaline phosphatase.

**Table 4:** The mediating effects of inflammatory factors on the association between log10-transformed Mefox and risk of COPD.

Variables	Indirect Effects	Direct Effects	Total Effects	Mediated Proportion (%)	*p*-Value
	β (95%CI)	β (95%CI)	β (95%CI)		
White blood cell (1000 cells/μL)	1.3181 (1.0038, 1.6323)	0.0211 (0.0083, 0.0339)	1.3392 (1.0247, 1.6537)	98.42	0.0012
Lymphocytes (1000 cells/μL)	−0.0227 (−0.2861, 0.2406)	0.0223 (0.0095, 0.0350)	−0.0004 (−0.2641, 0.2632)	4868.5	0.0006
Monocyte (1000 cells/μL)	0.1237 (0.1109, 0.1366)	0.0123 (−0.0007, 0.0253)	0.1361 (0.1178, 0.1543)	90.95	0.0628
Segmented neutrophils (1000 cells/μL)	1.1057 (0.9953, 1.2161)	0.0150 (0.0020, 0.0280)	1.1207 (1.0095, 1.2319)	98.66	0.0238
Eosinophils (1000 cells/μL)	0.1001 (0.0892, 0.1109)	0.0192 (0.0062, 0.0321)	0.1193 (0.1023, 0.1362)	83.93	0.0038
Basophils (1000 cells/μL)	0.0179 (0.0143, 0.0215)	0.0193 (0.0065, 0.0321)	0.0373 (0.0239, 0.0506)	48.18	0.0032
Red blood cell (million cells/μL)	−0.1350 (−0.1673, −0.1028)	0.0225 (0.0097, 0.0354)	−0.1125 (−0.1472, −0.0778)	120.04	0.0005
Hemoglobin (g/dL)	−0.2714 (−0.3710, −0.1718)	0.0224 (0.0096, 0.0352)	−0.2490 (−0.3494, −0.1486)	108.99	0.0006
Mean cell volume (fL)	0.4223 (0.0153, 0.8292)	0.0222 (0.0094, 0.0349)	0.4444 (0.0373, 0.8516)	95.01	0.0007
Mean cell Hgb concentration(g/dL)	0.1848 (0.1167, 0.2529)	0.0233 (0.0105, 0.0361)	0.2081 (0.1388, 0.2774)	88.79	0.0004
Red cell distribution width percent	0.1228 (0.0339, 0.2116)	0.0207 (0.0080, 0.0334)	0.1435 (0.0537, 0.2332)	85.56	0.0014
ALP (IU/L)	3.8530 (2.3387, 5.3673)	0.0201 (0.0073, 0.0329)	3.8731 (2.3587, 5.3874)	99.48	0.0020

MeFox, pyrazino-s-triazine derivative of 4α-hydroxy-5-methyltetrahydrofolate; CI, confidence interval. ALP, alkaline phosphatase.

## Data Availability

The data presented in this study are openly available on the NHANES website (https://www.cdc.gov/nchs/nhanes/ (accessed on 20 February 2024)).

## Supplementary Materials

The following supporting information can be downloaded at: https://www.mdpi.com/article/10.3390/healthcare14010040/s1, Table S1: Chi-square test for univariate risk of COPD. Table S2: Model I Multivariate logistic regression analysis of log10-transformed Mefox for risk of COPD. Table S3: Summary of statistical models, variables, and NHANES survey design handling.

## Abbreviations

Mefox: a pyrazino-s-triazine derivative of 4α-hydroxy-5-methyl-tetrahydrofolate; COPD, chronic obstructive pulmonary disease; NHANES, the National Health and Nutrition Examination Survey; RCS, Restricted cubic spline; CI, confidence interval; ROS, reactive oxygen species; 5-mTHF, 5-methyl-tetrahydrofolate; MECs, mobile examination centers; OR, odds ratio; BMI, body mass index; ALP, alkaline phosphatase; CDAI, Composite Dietary Antioxidant Index.
