# Supplementary material for: Association Between Mefox and Prevalence of COPD: Evidence from NHANES 2011–2020
Source: Healthcare (Basel). 2025 Dec 23;14(1):40. doi: 10.3390/healthcare14010040 (PMC12785568; doi:10.3390/healthcare14010040)
Supplement: Supplementary file 1 [file healthcare-14-00040-s001.zip › healthcare-3981871-supplementary.pdf]

healthcare

MDPI

Table S1. Chi-square test for univariate risk of COPD.

Variables	χ^2	P-value
log10-Mefox **(quartiles)**	10.419	0.0012473 **
Age	60.775	6.399e-15 ***
Gender	0.419	0.5174133
Race	2.117	0.1456449
Education level	26.005	3.405e-07 ***
BMI	0.112	0.7378240
Health insurance	1.132	0.2873268
Sedentary time	3.871	0.0491333 *
Alcohol	6.261	0.0123408 *
Smoked 100 cigarettes	116.139	< 2.2e-16 ***
High cholesterol	0.439	0.5076413
Diabetes	1.890	0.1692213
Hypertension	3.918	0.0477585 *
Coronary heart disease	13.478	0.0002414 ***
Stroke	11.861	0.0005732 ***
Cancer	2.637	0.1043917

log10-Mefox was transformed into an ordinal categorical variable by dividing it into quartiles (Q1–Q4) for descriptive chi-squared comparisons. MeFox, pyrazino-s-triazine derivative of 4α-hydroxy-5-methyltetrahydrofolate; BMI, body mass index. * $p < 0.05$, ** $p < 0.01$, *** $p < 0.001$.

Table S2. **Model I** Multivariate logistic regression analysis of log10-transformed Mefox for risk of COPD.

Variables	Estimate	Std. Error	Z value	OR 95% CI	P(>|z|)
log10-Mefox	0.14098	0.17488	0.806	1.15(0.82-1.62)	0.420145
Age	0.20472	0.04490	4.560	1.23(1.12-1.34)	5.13e-06 ***
Education level	-0.12591	0.04576	-2.752	0.88(0.81-0.96)	0.005929 **
Sedentary time	0.12662	0.07228	1.752	1.13(0.99-1.31)	0.079802
Alcohol	0.03362	0.11914	0.282	1.03(0.82-1.30)	0.777789
Smoked 100 cigarettes	-1.25442	0.13132	-9.553	0.29(0.22-0.37)	< 2e-16 ***
Hypertension	-0.19552	0.11403	-1.715	0.82(0.66-1.03)	0.086419
Coronary heart disease	-0.51637	0.17981	-2.872	0.60(0.42-0.84)	0.004082 **
Stroke	-0.69758	0.19082	-3.656	0.50(0.35-0.73)	0.000257 ***

Note: Model I was adjusted for log10-transformed Mefox and covariates primarily selected from variables that were significantly associated in Table S1, including age, education level, sedentary behavior, alcohol use, smoking, high blood pressure, coronary heart disease, and stroke. MeFox, pyrazino-s-triazine derivative of 4α-hydroxy-5-methyltetrahydrofolate; OR, odds ratio; CI, confidence interval. *$p < 0.05$, **$p < 0.01$, ***$p < 0.001$.

Table S3. Summary of statistical models, variables, and NHANES survey handling.

**Model**	**Outcome (Y)**	**Covariates included (detailed)**	**NHANES survey handling**
Crude logistic regression	COPD (yes/no)	Age; sex; race; education level; BMI; insurance; sedentary; alcohol; smoked ≥100 cigarettes; cholesterol; diabetes; hypertension; coronary heart disease; stroke; cancer.	Survey-weighted logistic regression (weights + strata + PSU)
Model I (adjusted logistic regression)	COPD (yes/no)	Age; education level; sedentary; alcohol; smoked ≥100 cigarettes; hypertension; coronary heart disease; stroke.	Survey-weighted logistic regression (weights + strata + PSU)

Model	Outcome (Y)	Covariates included (detailed)	NHANES survey handling
Restricted cubic spline (RCS) model	COPD (yes/no)	Same as Model I	Survey-weighted spline logistic regression (weights + strata + PSU)
Subgroup logistic regression	COPD (yes/no)	Same as crude Model	Survey-weighted within-subgroup models (weights + strata + PSU)
Mediation analysis	COPD (yes/no)	WBC, lymphocyte, monocyte, segmented neutrophil, eosinophil, and basophil, RBC, hemoglobin, MCV, MCHC, RDW, ALP.	A nonparametric bootstrapping framework

Note: PSU, Primary Sampling Unit; WBC, white blood cell; RBC, red blood cell; MCV, mean cell volume; MCHC, mean cell hemoglobin concentration; RDW, red cell distribution width; ALP, alkaline phosphatase.
